# Supplementary figures and images for: RB-TnSeq elucidates dicarboxylic-acid-specific catabolism in β-proteobacteria for improved plastic monomer upcycling
Source: Appl Environ Microbiol. 2025 Sep 22;91(10):e00924-25. doi: 10.1128/aem.00924-25 (PMC12543098; doi:10.1128/aem.00924-25)

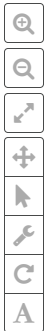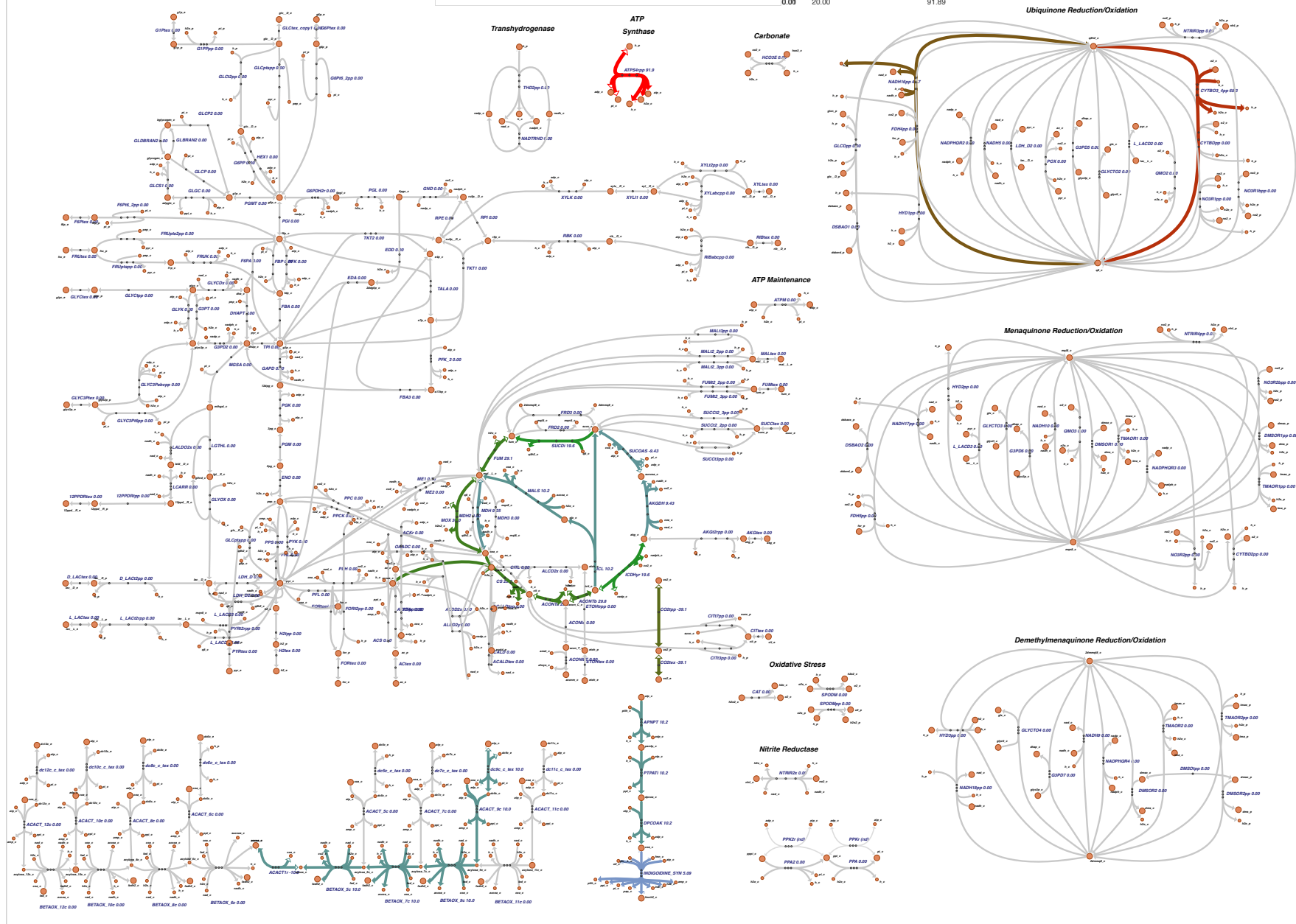

Supplement: File S5 — Escher figure of azelaic acid (C9DA) flux through modified metabolic model. [file aem.00924-25-s0005.pdf]
