# Supplementary material for: RB-TnSeq elucidates dicarboxylic-acid-specific catabolism in β-proteobacteria for improved plastic monomer upcycling
Source: Appl Environ Microbiol. 2025 Sep 22;91(10):e00924-25. doi: 10.1128/aem.00924-25 (PMC12543098; doi:10.1128/aem.00924-25)
Supplement: Supplemental figures — Figures S1 to S13. [file aem.00924-25-s0007.pdf]

Supplemental Figures: RB-TnSeq elucidates dicarboxylic acid specific catabolism in β-proteobacteria for improved plastic monomer upcycling

Allison N. Pearson[1,2,3], Julie M. Lynch[1], Cindy N. Ho[1], Graham A. Hudson[1,2,4,7], Jacob B. Roberts[1,2,5], Javier Menasalvas[1,2], Aaron A. Vilchez[1,2,9], Matthew R. Incha[1,2,3], Matthias Schmidt[1,2,6,7], Aindrila Mukhopadhyay[1,2,4], Adam M. Deutschbauer[3,4], Mitchell G. Thompson[1,4], Patrick M. Shih[1,3,4,8#], Jay D. Keasling[1,2,5,9,10#]

[1]Joint BioEnergy Institute, 5885 Hollis Street, Emeryville, CA 94608, USA.
[2]Biological Systems & Engineering Division, Lawrence Berkeley National Laboratory, Berkeley, CA 94720, USA.
[3]Department of Plant and Microbial Biology, University of California, Berkeley, CA 94720, USA
[4]Environmental Genomics and Systems Biology Division, Lawrence Berkeley National Laboratory, Berkeley, California, USA
[5]Joint Program in Bioengineering, University of California, Berkeley, California, USA
[6]Institute of Applied Microbiology (iAMB), Aachen Biology and Biotechnology (ABBt), RWTH Aachen University, Aachen, Germany
[7]California Institute for Quantitative Biosciences (QB3 Institute), University of California, Berkeley, CA, 94720, USA
[8]Innovative Genomics Institute, University of California, Berkeley, California, USA
[9]Department of Chemical and Biomolecular Engineering, University of California, Berkeley, CA 94720, USA
[10]The Novo Nordisk Foundation Center for Biosustainability, Technical University of Denmark, Denmark

#Corresponding authors : Jay D. Keasling, jdkeasling@lbl.gov; Patrick M. Shih pmshih@lbl.gov

Supplemental File Captions:

Supplemental File 1: Interactive version of Figure 2A: t-sne visualization of dicarboxylic acid fitness data.
Supplemental File 2: Interactive version of Figure S7B: sequence vs fitness similarity of regulators.
Supplemental File 3: Interactive version of Figure S7A: sequence vs fitness similarity of transporters.
Supplemental File 4: COBRA model for flux balance analysis.
Supplemental File 5: Escher metabolic map of azelaic acid (C9DA) flux through the modified metabolic model.
Supplemental File 6: Full RB-TnSeq datasets for all organisms.
Supplementary Tables: Supplementary Tables 1-15.

Supplemental Figures 1-13:

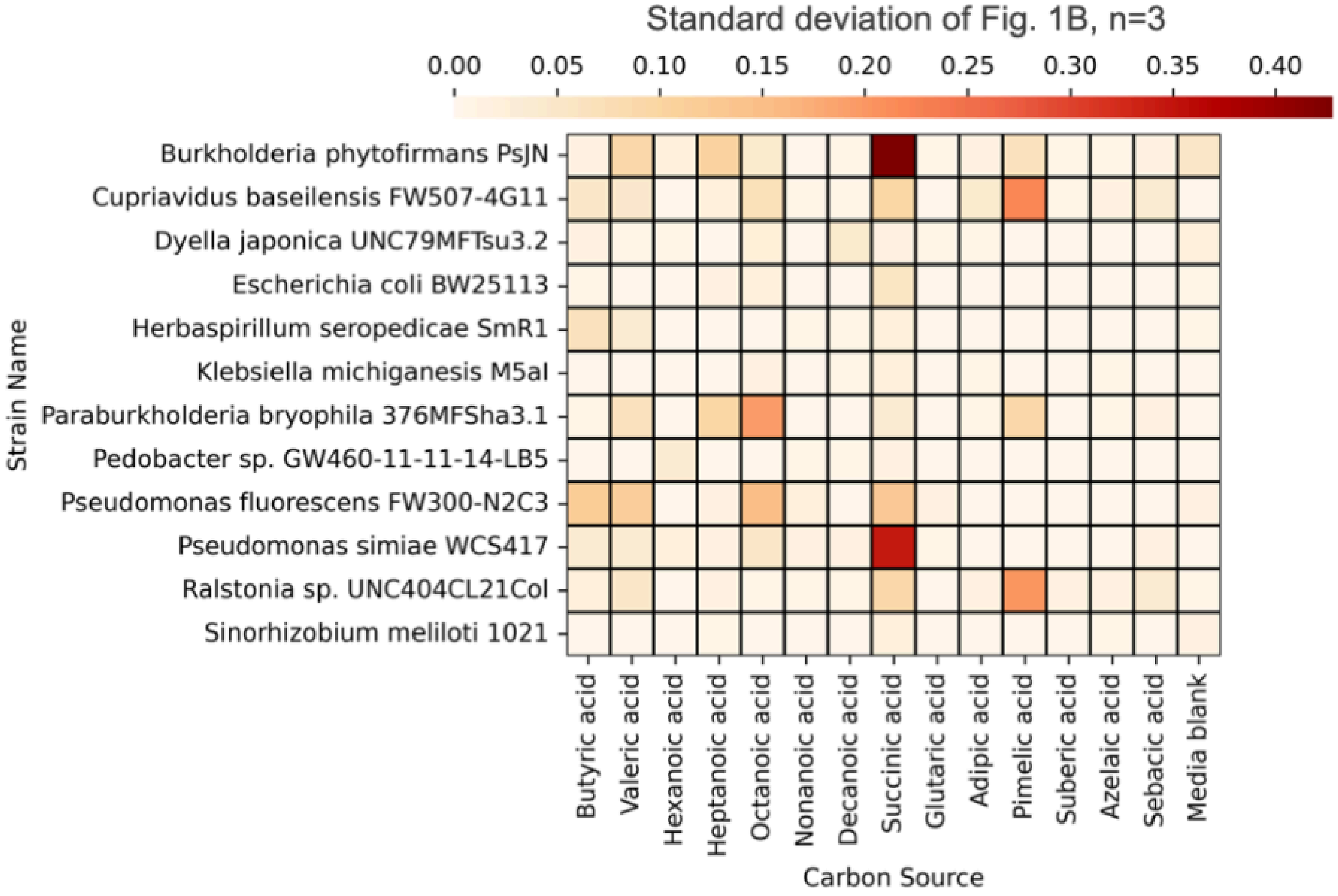

Figure S1: Heat map of error for Figure 1B (standard deviation, n=3)

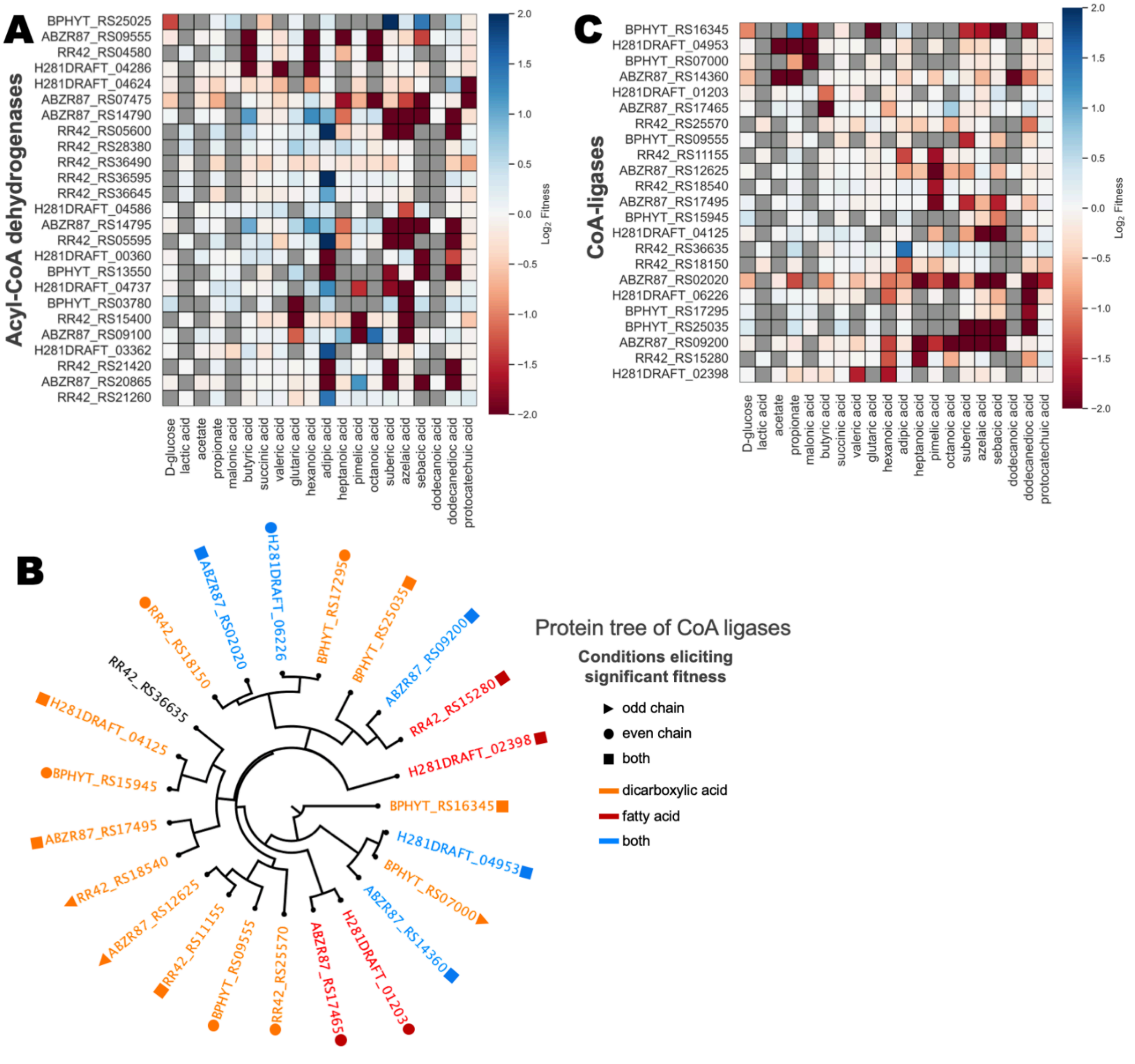

Figure S2: Fitness data for acyl-CoA dehydrogenases (A) and CoA ligases (B) with significant fitness (|fitness| > 1 and |t-score| > 4 and specific fitness on at least one of the experimental conditions. Genes are ordered to correspond with their respective phylogenetic trees (Figure 2D, Figure S2B). For conditions with two replicates that passed metric, the average fitness value is shown. Gray cells indicate that the library either did not grow or did not pass metric for that condition (see Supplementary Table 1). Acyl-CoA dehydrogenases were identified using the pFAMs listed in Supplementary Table 7. C) Phylogenetic tree of CoA ligase genes with significant fitness phenotypes. Genes with a negative fitness phenotype are colored according to which conditions elicit this phenotype (orange = dicarboxylic acid, red = fatty acid, and blue = both) and have a shape indication whether they have significant phenotypes for odd (triangle), even (circle), or both types of (square) carbon chains. The protein tree was constructed using MUSCLE with the super5 algorithm, and the UPGMA algorithm for tree construction. Tree is unrooted.

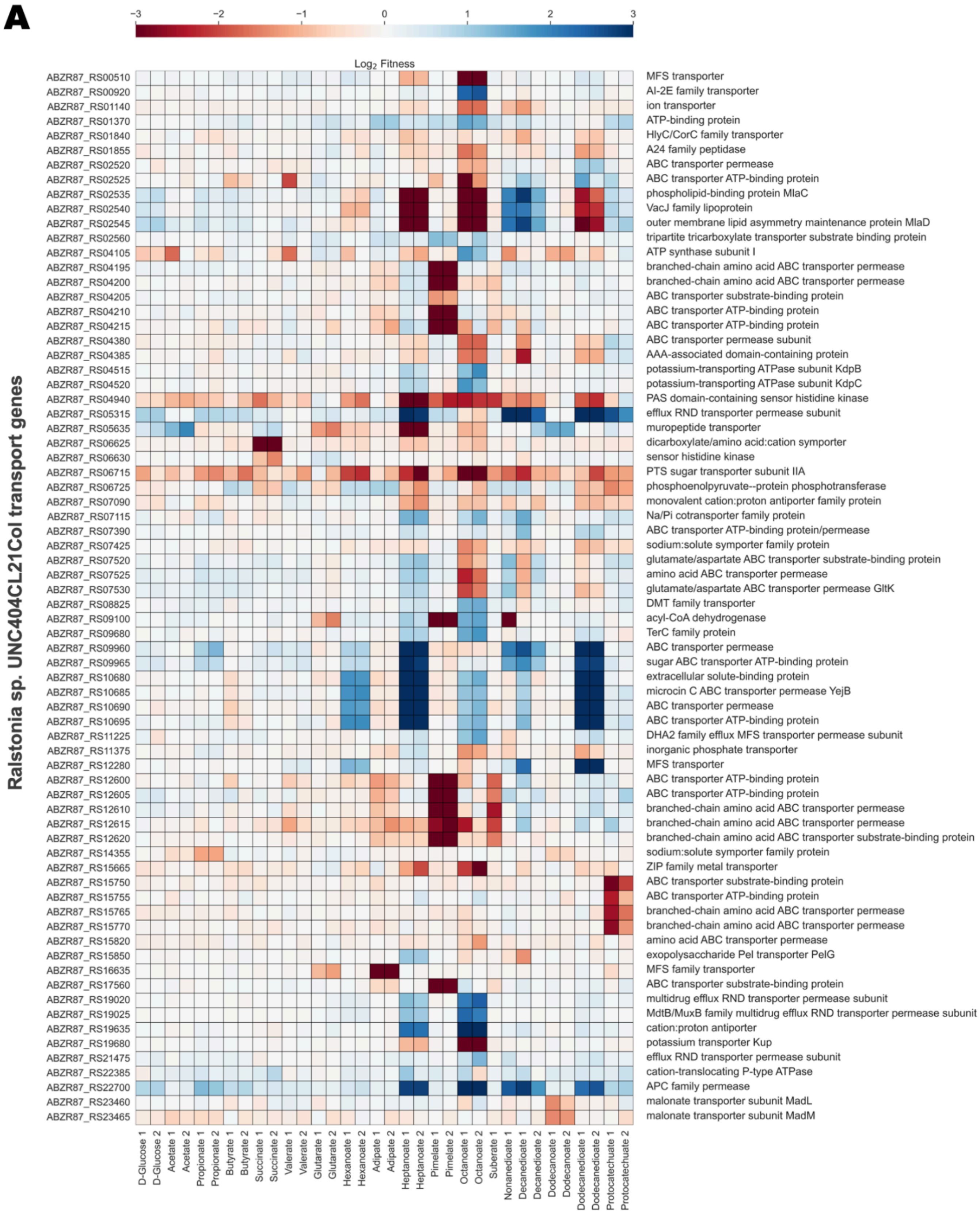
A
Log2 Fitness
−3
−2
−1
0
1
2
3
Ralstonia sp. UNC404CL21Col transport genes
ABZR87_RS00510 MFS transporter
ABZR87_RS00920 AI-2E family transporter
ABZR87_RS01140 ion transporter
ABZR87_RS01370 ATP-binding protein
ABZR87_RS01840 HlyC/CorC family transporter
ABZR87_RS01855 A24 family peptidase
ABZR87_RS02520 ABC transporter permease
ABZR87_RS02525 ABC transporter ATP-binding protein
ABZR87_RS02535 phospholipid-binding protein MlaC
ABZR87_RS02540 VacJ family lipoprotein
ABZR87_RS02545 outer membrane lipid asymmetry maintenance protein MlaD
ABZR87_RS02560 tripartite tricarboxylate transporter substrate binding protein
ABZR87_RS04105 ATP synthase subunit I
ABZR87_RS04195 branched-chain amino acid ABC transporter permease
ABZR87_RS04200 branched-chain amino acid ABC transporter permease
ABZR87_RS04205 ABC transporter substrate-binding protein
ABZR87_RS04210 ABC transporter ATP-binding protein
ABZR87_RS04215 ABC transporter ATP-binding protein
ABZR87_RS04380 ABC transporter permease subunit
ABZR87_RS04385 AAA-associated domain-containing protein
ABZR87_RS04515 potassium-transporting ATPase subunit KdpB
ABZR87_RS04520 potassium-transporting ATPase subunit KdpC
ABZR87_RS04940 PAS domain-containing sensor histidine kinase
ABZR87_RS05315 efflux RND transporter permease subunit
ABZR87_RS05635 muropeptide transporter
ABZR87_RS06625 dicarboxylate/amino acid:cation symporter
ABZR87_RS06630 sensor histidine kinase
ABZR87_RS06715 PTS sugar transporter subunit IIA
ABZR87_RS06725 phosphoenolpyruvate--protein phosphotransferase
ABZR87_RS07090 monovalent cation:proton antiporter family protein
ABZR87_RS07115 Na/Pi cotransporter family protein
ABZR87_RS07390 ABC transporter ATP-binding protein/permease
ABZR87_RS07425 sodium:solute symporter family protein
ABZR87_RS07520 glutamate/aspartate ABC transporter substrate-binding protein
ABZR87_RS07525 amino acid ABC transporter permease
ABZR87_RS07530 glutamate/aspartate ABC transporter permease GltK
ABZR87_RS08825 DMT family transporter
ABZR87_RS09100 acyl-CoA dehydrogenase
ABZR87_RS09680 TerC family protein
ABZR87_RS09960 ABC transporter permease
ABZR87_RS09965 sugar ABC transporter ATP-binding protein
ABZR87_RS10680 extracellular solute-binding protein
ABZR87_RS10685 microcin C ABC transporter permease YejB
ABZR87_RS10690 ABC transporter permease
ABZR87_RS10695 ABC transporter ATP-binding protein
ABZR87_RS11225 DHA2 family efflux MFS transporter permease subunit
ABZR87_RS11375 inorganic phosphate transporter
ABZR87_RS12280 MFS transporter
ABZR87_RS12600 ABC transporter ATP-binding protein
ABZR87_RS12605 ABC transporter ATP-binding protein
ABZR87_RS12610 branched-chain amino acid ABC transporter permease
ABZR87_RS12615 branched-chain amino acid ABC transporter permease
ABZR87_RS12620 branched-chain amino acid ABC transporter substrate-binding protein
ABZR87_RS14355 sodium:solute symporter family protein
ABZR87_RS15665 ZIP family metal transporter
ABZR87_RS15750 ABC transporter substrate-binding protein
ABZR87_RS15755 ABC transporter ATP-binding protein
ABZR87_RS15765 branched-chain amino acid ABC transporter permease
ABZR87_RS15770 branched-chain amino acid ABC transporter permease
ABZR87_RS15820 amino acid ABC transporter permease
ABZR87_RS15850 exopolysaccharide Pel transporter PelG
ABZR87_RS16635 MFS family transporter
ABZR87_RS17560 ABC transporter substrate-binding protein
ABZR87_RS19020 multidrug efflux RND transporter permease subunit
ABZR87_RS19025 MdtB/MuxB family multidrug efflux RND transporter permease subunit
ABZR87_RS19635 cation:proton antiporter
ABZR87_RS19680 potassium transporter Kup
ABZR87_RS21475 efflux RND transporter permease subunit
ABZR87_RS22385 cation-translocating P-type ATPase
ABZR87_RS22700 APC family permease
ABZR87_RS23460 malonate transporter subunit MadL
ABZR87_RS23465 malonate transporter subunit MadM
D-Glucose 1
D-Glucose 2
Acetate 1
Acetate 2
Propionate 1
Propionate 2
Butyrate 1
Butyrate 2
Succinate 1
Succinate 2
Valerate 1
Valerate 2
Glutarate 1
Glutarate 2
Hexanoate 1
Hexanoate 2
Adipate 1
Adipate 2
Heptanoate 1
Heptanoate 2
Pimelate 1
Pimelate 2
Octanoate 1
Octanoate 2
Suberate 1
Nonanedioate 1
Decanedioate 1
Decanedioate 2
Dodecanoate 1
Dodecanoate 2
Dodecanedioate 1
Dodecanedioate 2
Protocatechuate 1
Protocatechuate 2

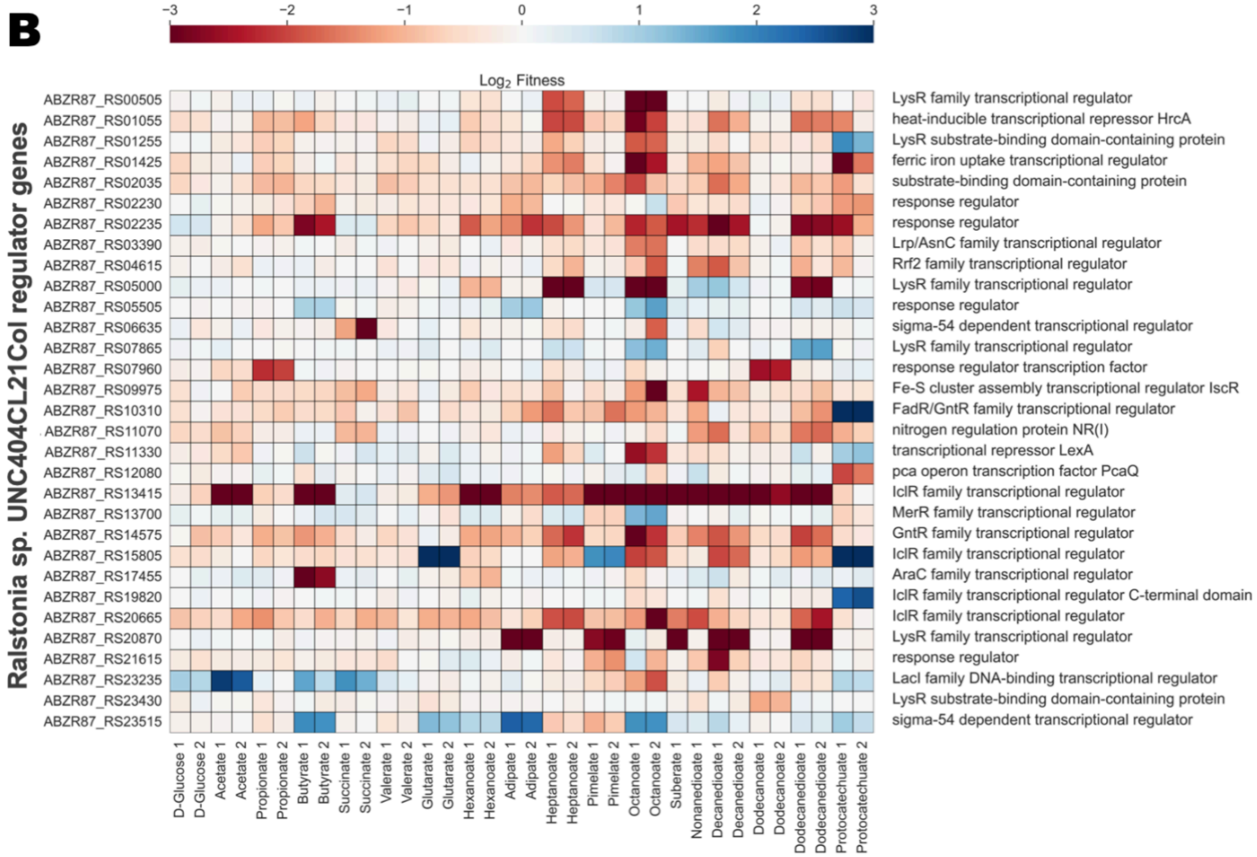

Figure S3: Fitness data for A) predicted transport genes and B) predicted regulatory genes with significant fitness (|fitness| > 1 and |t-score| > 4) and specific fitness on at least one of the experimental conditions in *R. CL21*. Transport genes were identified with TransAAP and regulatory genes were identified using the pFAMs shown in Supplementary Tables 3 and 4.

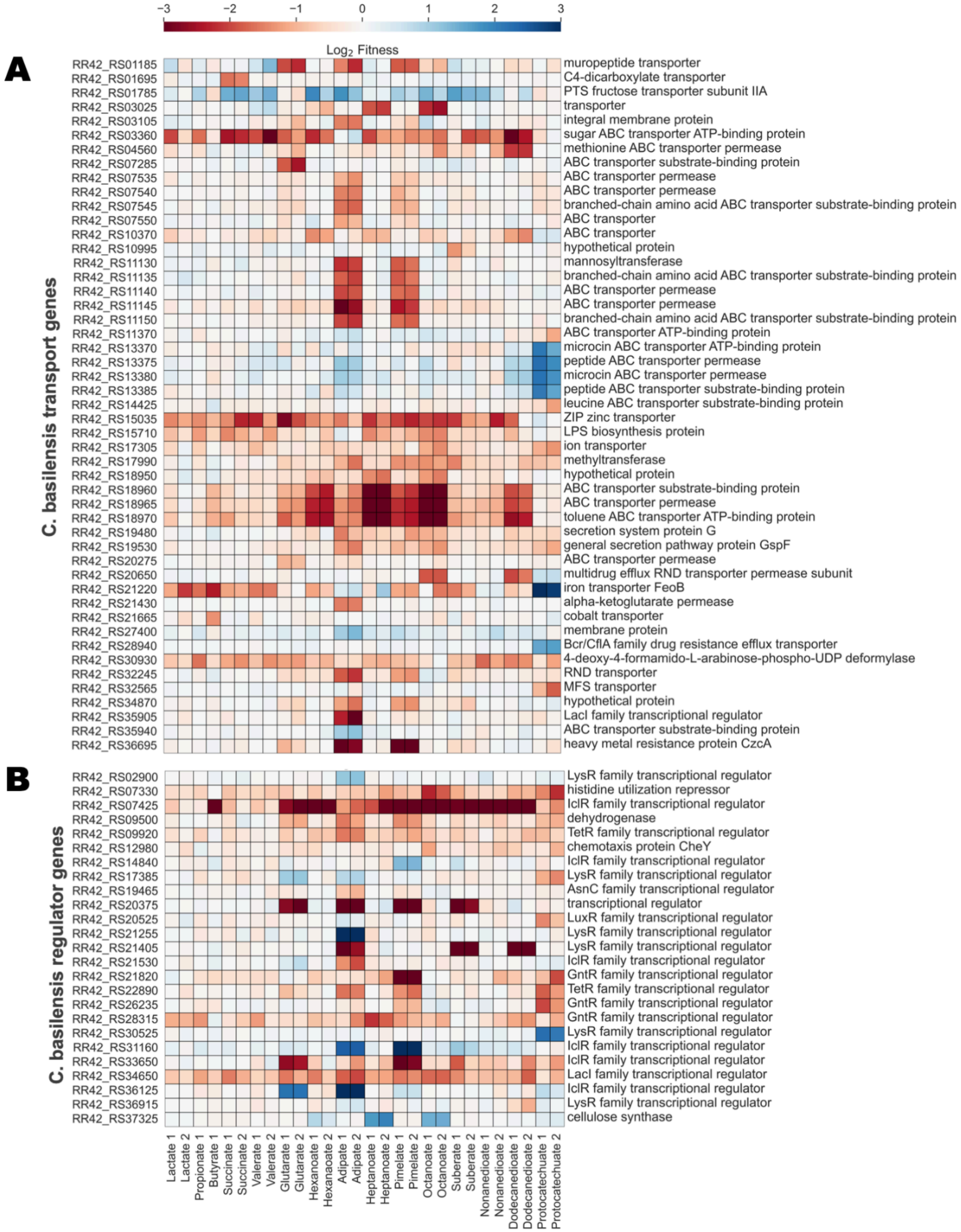

Figure S4: Fitness data for A) predicted transport genes and B) predicted regulatory genes with significant fitness (|fitness| > 1 and |t-score| > 4) and specific fitness on at least one of the experimental conditions in *C. basilensis*. Transport genes were identified with TransAAP and regulatory genes were identified using the pFAMs shown in Supplementary Tables 3 and 4.

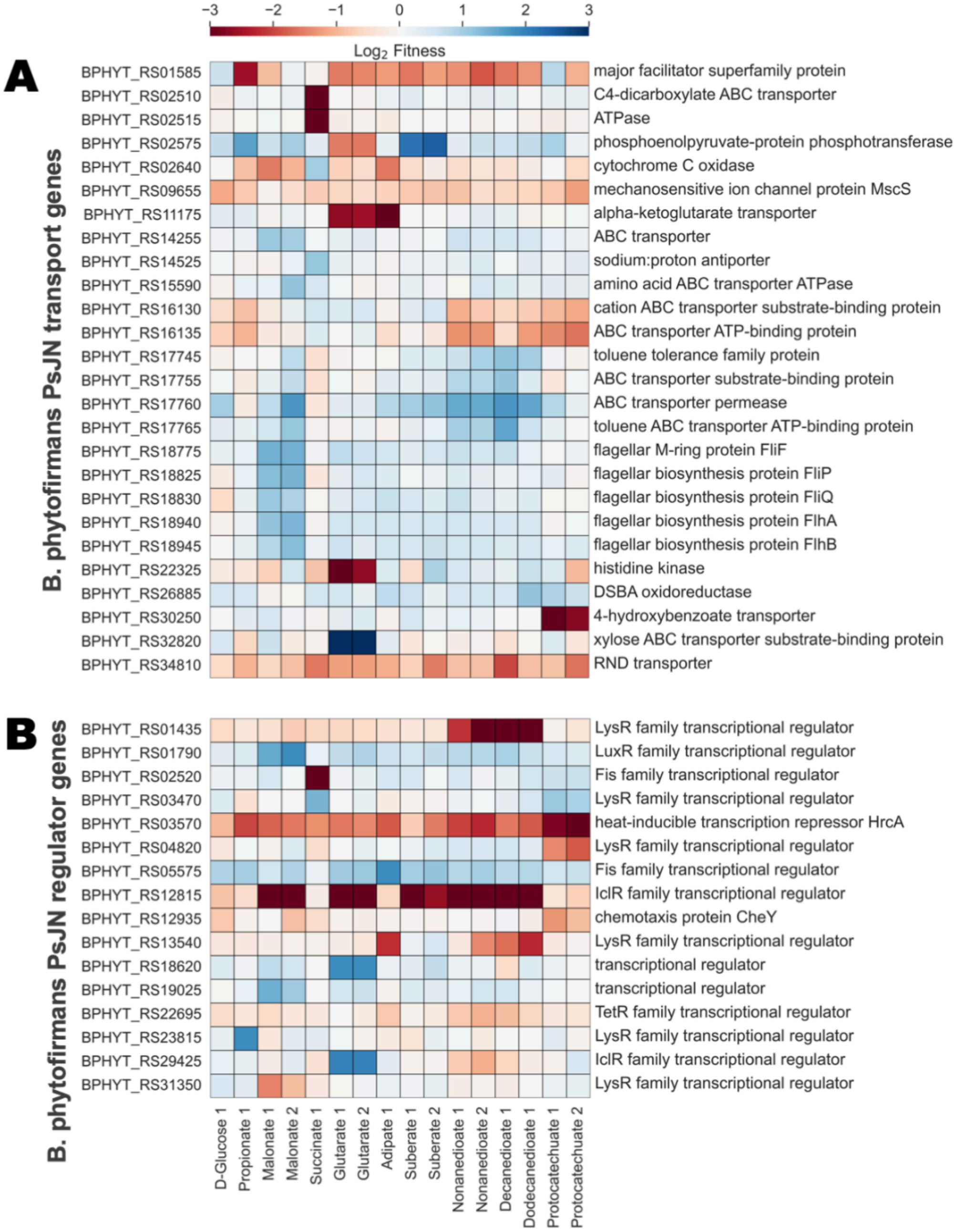

Figure S5: Fitness data for A) predicted transport genes and B) predicted regulatory genes with significant fitness (|fitness| > 1 and |t-score| > 4) and specific fitness on at least one of the experimental conditions in *B. phytofirmans*. Transport genes were identified with TransAAP and regulatory genes were identified using the pFAMs shown in Supplementary Tables 3 and 4.

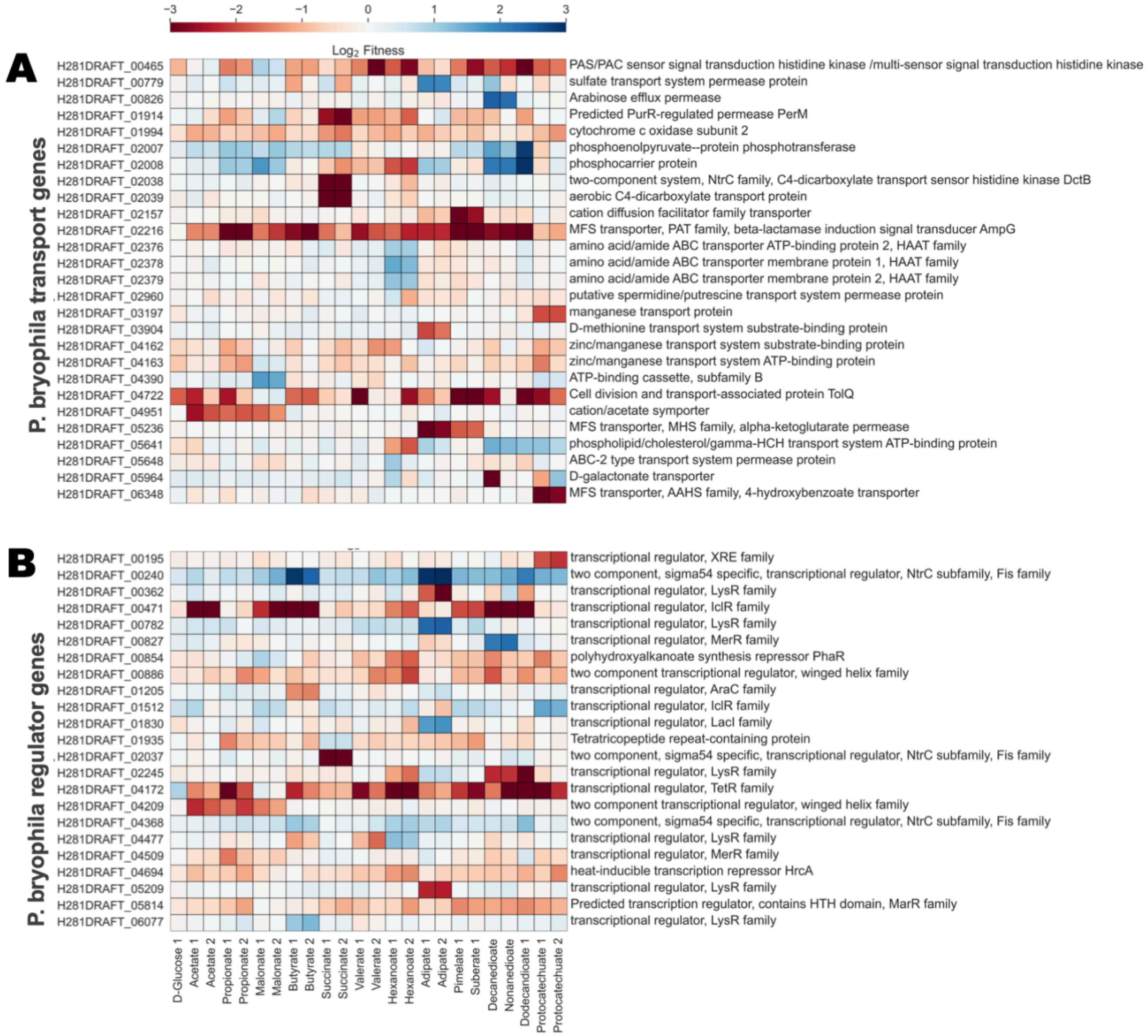

Figure S6: Fitness data for A) predicted transport genes and B) predicted regulatory genes with significant fitness (|fitness| > 1 and |t-score| > 4) and specific fitness on at least one of the experimental conditions in *P. bryophila*. Transport genes were identified with TransAAP and regulatory genes were identified using the pFAMs shown in Supplementary Tables 3 and 4.

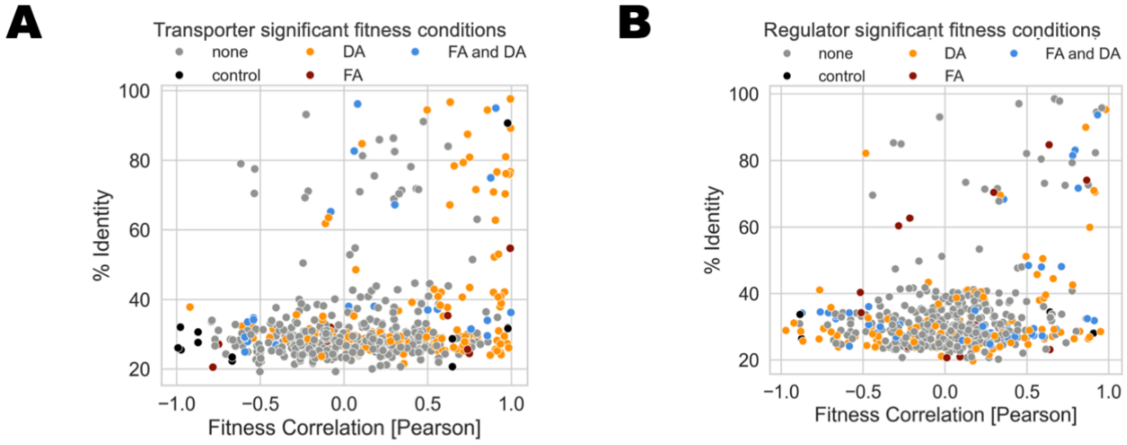

Figure S7: Correlation versus % identity in A) transport genes and B) regulatory genes. Pairwise BLASTp with an e-value cutoff of 0.001 was performed on all genes with a significant phenotype, along with a Pearson correlation of their fitness profiles across all conditions. Each point represents a pairwise comparison between genes. Points are colored by whether the two genes they represent have significant phenotypes on either dicarboxylic acids (orange), fatty acids (red), fatty and dicarboxylic acids (blue), the control protocatechuate and acetate conditions (black), or don't share any of these categories (grey). An interactive version can be found in Supplemental Files 2 and 3.

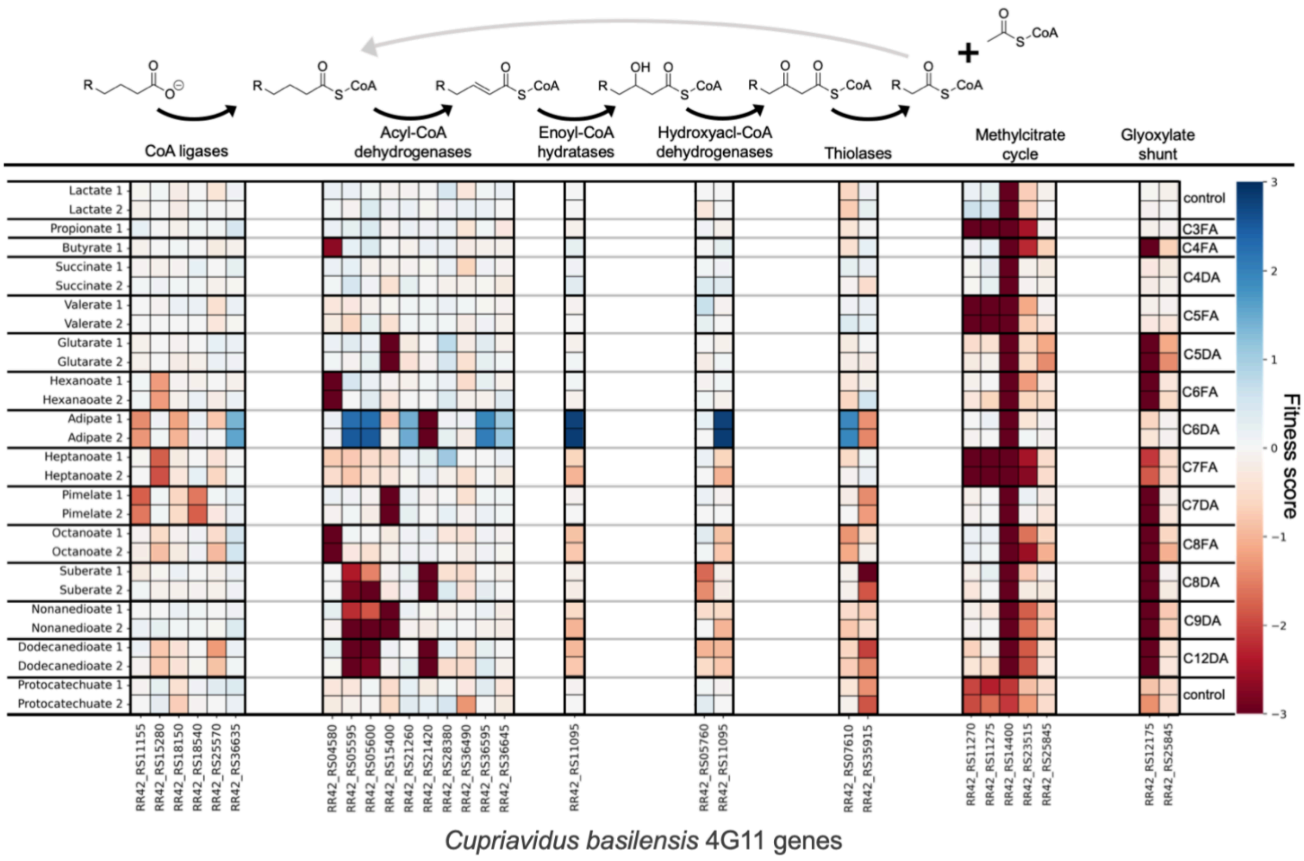

Figure S8: RB-TnSeq data for genes involved in β-oxidation in *C. basilensis.* A diagram of one round of β-oxidation is shown at the top. Below, we show the genes predicted by pFAM to encode for β-oxidation enzymes, the glyoxylate shunt, and the methylcitrate cycle that had significant (|fitness| > 1 and |t-score| > 4) fitness phenotypes, and the corresponding fitness values on the conditions tested.

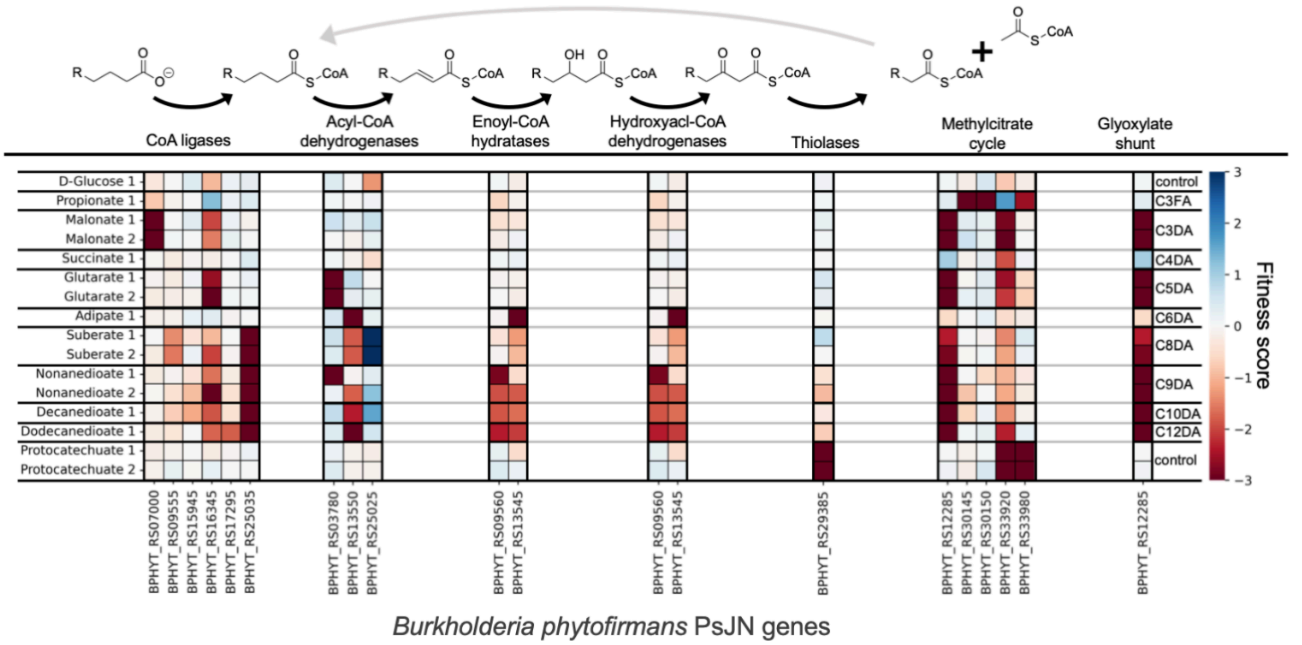

Figure S9: RB-TnSeq data for genes involved in β-oxidation in *B. phytofirmans.* A diagram of one round of β-oxidation is shown at the top. Below, we show the genes predicted by pFAM to encode for β-oxidation enzymes, the glyoxylate shunt, and the methylcitrate cycle that had significant (|fitness| > 1 and |t-score| > 4) fitness phenotypes, and the corresponding fitness values on the conditions tested.

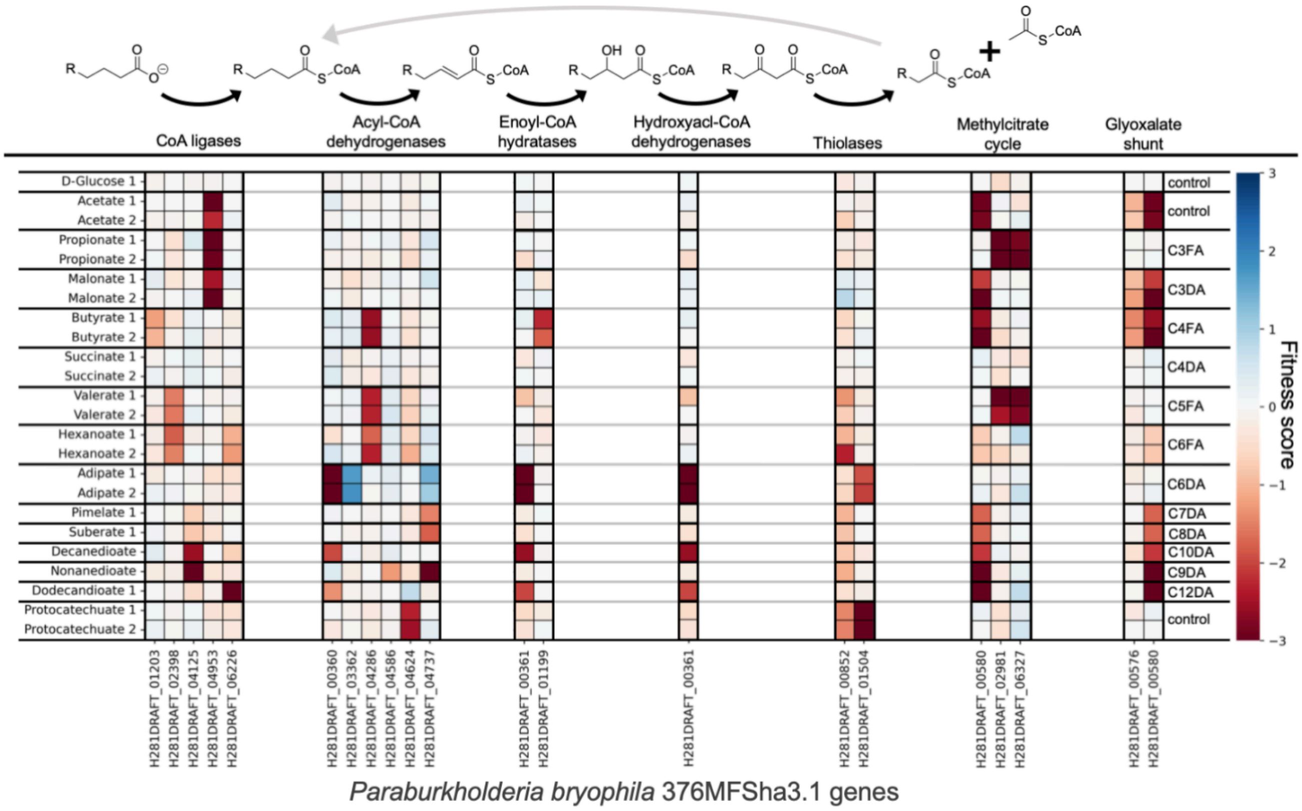

Figure S10: RB-TnSeq data for genes involved in β-oxidation in *P. bryophila.* A diagram of one round of β-oxidation is shown at the top. Below, we show the genes predicted by pFAM to encode for β-oxidation enzymes, the glyoxylate shunt, and the methylcitrate cycle that had significant (|fitness| > 1 and |t-score| > 4) fitness phenotypes, and the corresponding fitness values on the conditions tested.

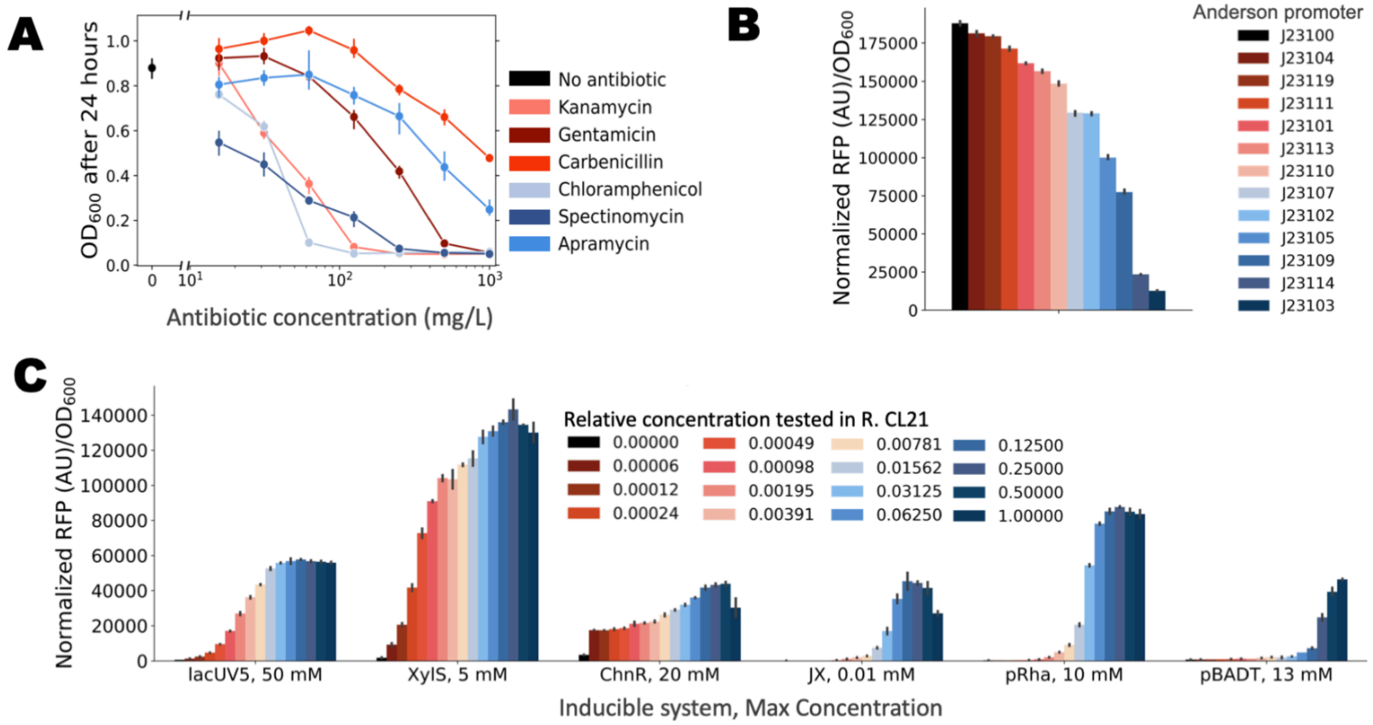

Figure S11: A) Optical density of *R. CL21* grown in the presence of seven antibiotics to calculate minimum inhibitory concentration in *R. CL21* (n=3, error bars = 95% confidence interval). Of the antibiotics tested, only kanamycin, chloramphenicol, and high concentrations of spectinomycin were efficient at inhibiting the growth of *R. CL21*. For the work we performed, 30 mg/mL gentamicin was typically selected for strain maintenance and 300 mg/mL kanamycin was used as a selective marker. B) RFP expression from constitutive promoters in *R. CL21* (n=3, error = 95% confidence interval). C) RFP expression from inducible systems in *R. CL21*. Max inducer concentration tested for each system is stated on the x-axis, color hue indicates relative concentration

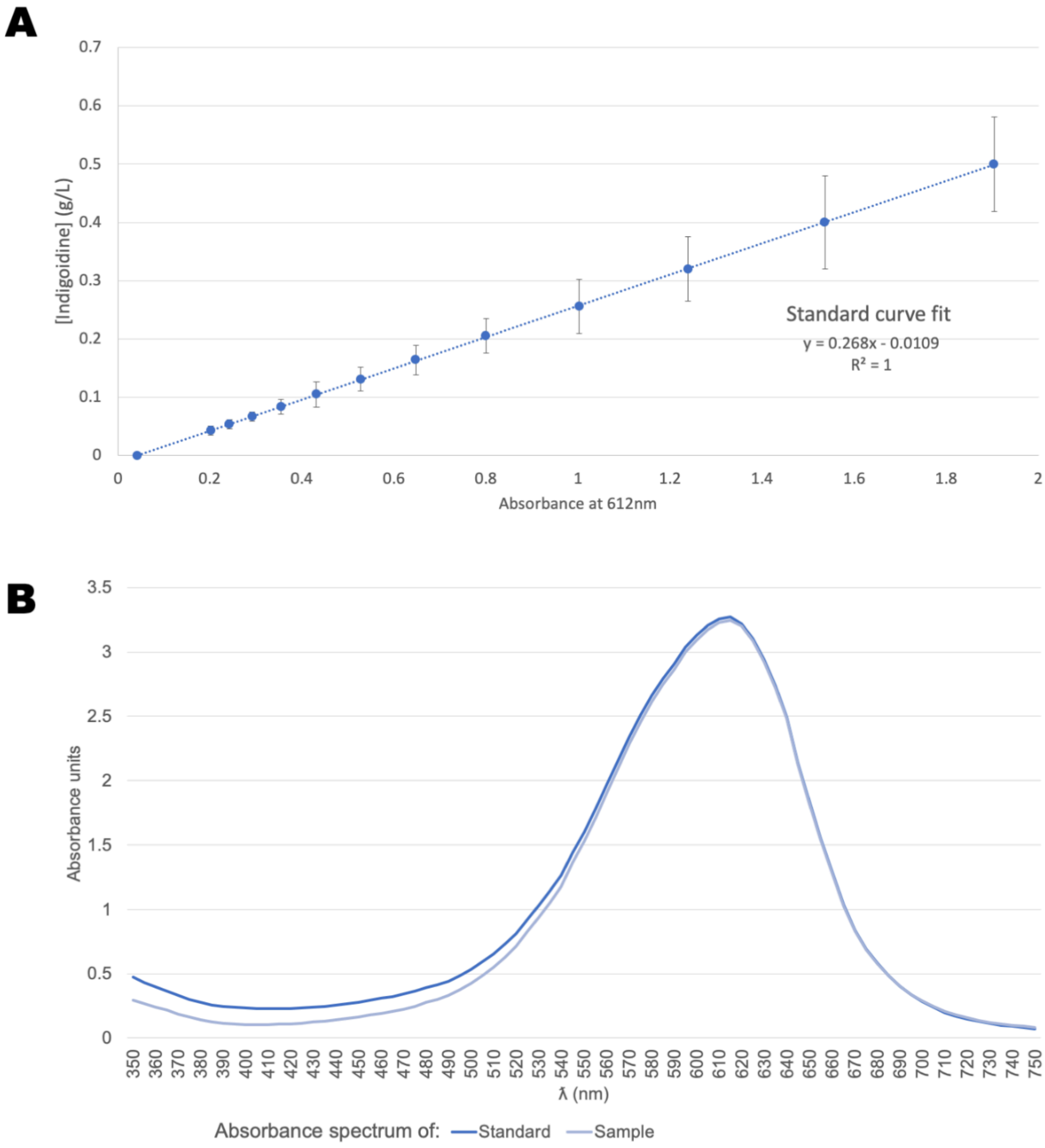

Figure S12: A) Indigoidine standard curve. (n = 3, error = standard deviation). B) Absorbance spectra of indigoidine standard and sample, dissolved in DMSO. Pure DMSO was used as a blank.

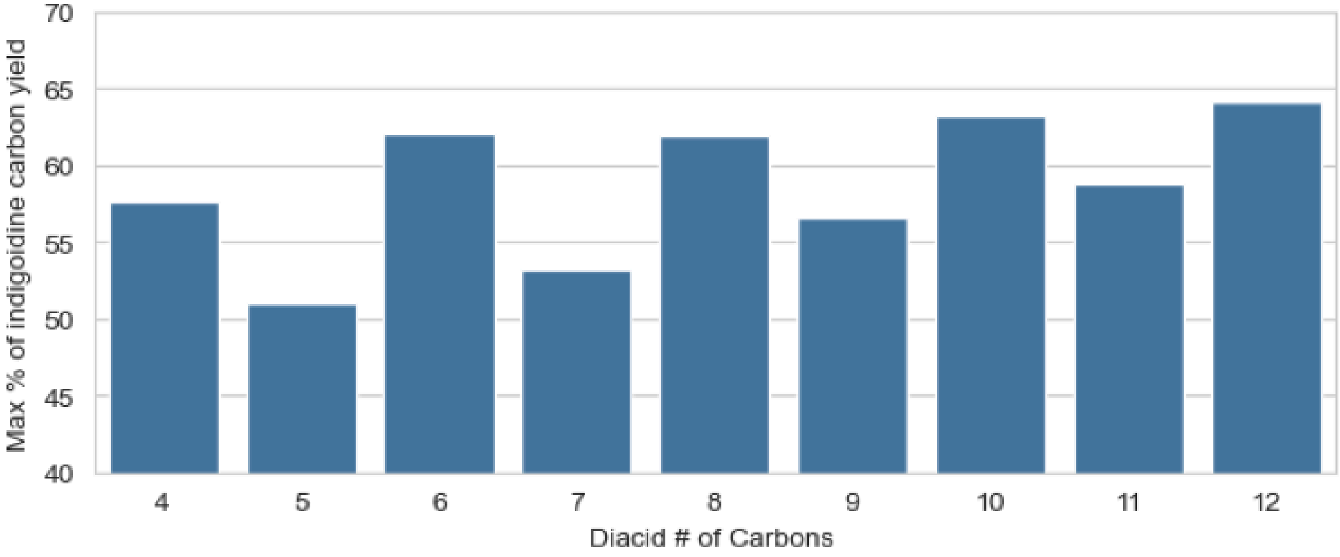

Figure S13: Breakdown of maximum percentage of carbon yield estimation with each individual dicarboxylic acid carbon source.
